# Predicting survival of pancreatic cancer patients treated with gemcitabine using longitudinal tumour size data

**DOI:** 10.1007/s00280-016-2994-x

**Published:** 2016-03-03

**Authors:** Thierry Wendling, Hitesh Mistry, Kayode Ogungbenro, Leon Aarons

**Affiliations:** Manchester Pharmacy School, The University of Manchester, Stopford Building Room 3.32, Oxford Road, Manchester, M13 9PT UK; Drug Metabolism and Pharmacokinetics, Novartis Institutes for Biomedical Research, 4002 Basel, Switzerland

**Keywords:** Metastatic pancreatic cancer, Gemcitabine, Tumour size time-series, Hierarchical modelling, Survival analysis

## Abstract

**Purpose:**

Measures derived from longitudinal tumour size data have been increasingly utilised to predict survival of patients with solid tumours. The aim of this study was to examine the prognostic value of such measures for patients with metastatic pancreatic cancer undergoing gemcitabine therapy.

**Methods:**

The control data from two Phase III studies were retrospectively used to develop (271 patients) and validate (398 patients) survival models. Firstly, 31 baseline variables were screened from the training set using penalised Cox regression. Secondly, tumour shrinkage metrics were interpolated for each patient by hierarchical modelling of the tumour size time-series. Subsequently, survival models were built by applying two approaches: the first aimed at incorporating model-derived tumour size metrics in a parametric model, and the second simply aimed at identifying empirical factors using Cox regression. Finally, the performance of the models in predicting patient survival was evaluated on the validation set.

**Results:**

Depending on the modelling approach applied, albumin, body surface area, neutrophil, baseline tumour size and tumour shrinkage measures were identified as potential prognostic factors. The distributional assumption on survival times appeared to affect the identification of risk factors but not the ability to describe the training data. The two survival modelling approaches performed similarly in predicting the validation data.

**Conclusions:**

A parametric model that incorporates model-derived tumour shrinkage metrics in addition to other baseline variables could predict reasonably well survival of patients with metastatic pancreatic cancer. However, the predictive performance was not significantly better than a simple Cox model that incorporates only baseline characteristics.

**Electronic supplementary material:**

The online version of this article (doi:10.1007/s00280-016-2994-x) contains supplementary material, which is available to authorized users.

## Introduction

Pancreatic cancer is a common cause of cancer-related death and is difficult to treat as diagnosis is often made late and patients present with metastatic disease [1]. Despite recent improvements in diagnostic techniques, the prognosis of patients with pancreatic cancer is poor, with a 5-year survival rate of 0.4–4 % [2]. The only potential curative treatment is surgical resection although only 15–20 % of patients are eligible for surgery [1]. Gemcitabine chemotherapy is the standard palliative care [3], with a median survival of 5.7 months and 20 % 1-year survival rate [4]. However, the prognosis of patients receiving palliative chemotherapies varies depending on their clinical characteristics [5]. It is therefore important to identify subgroups of patients that would benefit from chemotherapies. Several prognostic factors for patients with metastatic pancreatic cancer (MPC) have been previously identified: pancreatic cancer location [6], albumin level (ALB), carbohydrate antigen 19-9 level (CA19-9), alkaline phosphatase level (ALP), lactate dehydrogenase level, white blood cell count, aspartate aminotransferase level, blood urea nitrogen level [7], long-standing diabetes [8], Eastern Cooperative Oncology Group (ECOG) performance status, C-reactive protein level [9], the status of unresectable disease, carcinoembryonic antigen level and neutrophil–lymphocyte ratio [10].

Identifying prognostic factors with predictive value for patient risk stratification is an important task in cancer research to monitor and assess clinical trials, and individualise patient care. Multivariate statistical modelling methods are commonly used to investigate survival in relation to a factor of interest (e.g. treatment exposure) while adjusting for others (e.g. genotype). The Cox proportional hazards (CPH) model [11] is the most frequently used multivariate regression method, mainly because no assumption on the distribution of survival times is required (“semi-parametric” method) although it relies on the key assumption that the group-specific hazards are proportional over time. A useful alternative when the proportional hazards assumption does not hold is to use the accelerated failure time (AFT) model [12] which offers the advantage of an easier interpretation of the covariate effects (directly on the survival time) as compared to the CPH model (effects on the hazard rate). Since the AFT model is fully parametric, it is also more suitable for simulations that can be of value to extrapolate patient survival data and to optimise the design of oncology clinical trials. Nevertheless, the assumption on the survival time distribution might be deemed too strict as most of the time the distribution (or even a close approximation) is unknown.

In oncology drug development, decision-making and trial design are traditionally based on the Response Evaluation Criteria In Solid Tumours (RECIST), an empirical categorical measure of antitumor activity [13, 14]. More recently, several studies have shown that model-derived tumour size (TS) metrics can also be used as predictors for survival of patients with solid tumours [15–18]. In drug development, a parametric survival model that includes early change in TS as predictor variables might be beneficial for trial design and early assessment of drug efficacy. To our knowledge, this approach has not been evaluated in MPC.

The objective of this study is to apply a fully parametric drug development approach (referred to as the “PAR approach”) that utilises longitudinal TS data, to predict the mortality risk of patients with MPC. We used the control arm data from two Phase III MPC studies to build and validate the models as it would be done in the clinic. Early changes in TS were interpolated by hierarchical nonlinear modelling of the TS time-series and were tested as predictors for survival in an AFT model. We also compared the predictive performance between the PAR approach and a more conventional clinical approach that uses a CPH regression of empirical risk factors.

## Materials and methods

### Data

The models were built on the control arm data of a Phase III study in which 546 patients with MPC were randomly assigned to receive either gemcitabine (*n* = 275) or aflibercept (*n* = 271) [19]. The predictive performance of the models was then evaluated using an independent (validation) data set from the control arm of a Phase III study of 861 patients with MPC randomly assigned to nab-paclitaxel plus gemcitabine (*n* = 431) or gemcitabine (*n* = 430) [20]. Only gemcitabine data (control arms) were available for the present analysis. Although our work was done retrospectively, the choice of the training and validation sets was based on the chronology of the clinical studies in order to mimic the time constraints of statistical analyses in clinical research. Details of patient characteristics and the study designs can be found in the original study reports [19, 20]. The primary endpoint in both studies was overall survival, defined as the time from randomisation to the time of death from any cause. We instead used as model outcome the survival time defined as the time from randomisation to the time of death from the disease. Event times and baseline clinical characteristics were available for 271 patients in the training set and for 398 patients in the validation set. Censored times were extracted from the date of the last visit (for assessment of tumour response or laboratory variables). Among the patients who died in the training set (*n* = 147) and in the validation set (*n* = 370), only 13 and 8 %, respectively, died from other causes than cancer progression. In both studies, antitumor activity was evaluated every 8 weeks according to RECIST version 1.0 criteria [14].

### Statistical analysis

Firstly, potential risk factors were screened among previously reported prognostic factors and patient clinical characteristics from the training set. Secondly, TS reduction metrics were interpolated at relevant times for each patient of both the training and validation sets, by modelling separately the TS time-series from the two data sets. Subsequently, we developed a multivariate survival model using two different approaches: the PAR approach that aims at relating model-derived TS reduction metrics to survival time using the AFT model, and a more classic clinical approach that aims at identifying empirical risk factors using a Cox regression model (referred to as the “COX approach”). Finally, the performance of these two approaches in predicting patient mortality risk was compared using the validation set.

#### Screening risk factors

Thirty-one prognostic factors or baseline clinical characteristics for all 271 patients of the training set (128 death from disease) were screened to identify potential survival predictors: gender, race, age, body surface area (BSA), body weight, body height, ECOG status, cancer location (CLOC, defined categorically as entire pancreas or pancreas head versus pancreas tail or pancreas body), stage of cancer (from I to IV), diabetes status (yes or no), smoking status (yes or no), baseline TS, ALB, ALP, alanine transaminase, aspartate aminotransferase, bilirubin level (BILI), calcium, CA19-9, creatinine, glucose, haemoglobin, potassium, magnesium, neutrophil count (NEUT), phosphorus, platelets, total protein, white blood cell count and sodium levels. Details on the distribution of these variables can be found in the original study report. The ALP, CA19-9 and NEUT variables were log-transformed as their distributions in the studied patients are highly skewed. Twenty-one variables had <30 % of missing values which were multiply imputed using complete cases of all variables including the response variable (death status) [21].

Variable selection was done using a CPH regression with the least absolute shrinkage and selection operator (LASSO) penalty [22]. Using LASSO regularisation, the coefficients of the variables that are apparently not important shrink to zero, thereby producing a sparse model. Since the number of death events was small (*n* = 128), the algorithm was forced to retain no more than 10 variables in the sparse model. All continuous variables were standardised prior to the regression as they were not in the same units. The optimal value of the penalty tuning parameter was determined by *k*-fold cross-validation using the model partial-likelihood deviance as loss function. Regardless of the results, factors deemed clinically relevant (e.g. biomarker or established risk factors) were evaluated in the subsequent survival analysis aiming at quantifying the predictive value of the selected factors and of early change in TS metrics.

#### Modelling tumour size time-series

TS measures were derived as the sum of the longest diameters of the target lesions based on the RECIST criteria. In order to further compare empirical and model-derived early changes in TS metrics as survival predictors, only patients who had a pre-treatment and at least one on-treatment TS assessment were included in the time-series analysis (152 and 385 patients for the training and validation sets, respectively). To account for random interpatient and residual variability in the data, a three-stage hierarchical model was developed. At the first stage, the model likelihood was assumed lognormally distributed. A mixed model with exponential-decay and linear-growth components was used to describe the time course of TS change, using the same nomenclature as Wang et al. [15] (Eq. 1).

1 $${\text{TS}}_{i} (t) = {\text{BASE}}_{i} \cdot e^{{ - {\text{SR}}_{i} \cdot t}} + {\text{PR}}_{i} \cdot t$$

In Eq. 1, ${\text{TS}}_{i} (t)$ is the TS at time *t* for the *i*th patient, ${\text{BASE}}_{i}$ is the baseline TS, ${\text{SR}}_{i}$ is the decay or tumour shrinkage rate constant, and ${\text{PR}}_{i}$ is the regrowth or tumour progression rate constant. At the second stage, we assumed that each parameter follows a lognormal distribution in the patient population. For example, the distribution of the baseline TS was defined as follows:

2 $${\text{BASE}}_{i} = e^{{\theta_{\text{BASE}} + \eta_{{{\text{BASE}},i}} }}$$

where $\theta_{\text{BASE}}$ is the population mean of the log-transformed parameter and $\eta_{{{\text{BASE}},i}}$ is the difference between the individual and the population mean log-transformed values that is assumed normally distributed with mean 0 and standard deviation $\omega_{\text{BASE}} .$ Finally at the third stage, we assigned uninformative prior distributions to the model parameters as well as to the residual standard deviation $\sigma$ (see Stan code in “Appendix 1”).

Sampling from the posterior distribution of the parameters was done using the No-U-Turn variant of the Hamiltonian Monte Carlo algorithm [23], as implemented in the software Stan [24]. Convergence to approximate equilibrium was monitored using the potential scale reduction statistics [25]. Since the purpose of the modelling was to interpolate TS for each patient, the main diagnostic criterion for the model was its ability to capture observed individual profiles. We then used the posterior mean of the individual parameters to predict for each patient the percentage TS change from baseline (PTR) at early time points, i.e. 2, 4, 6, 8 and 10 weeks, in order to further evaluate the predictive value of these model-derived TS metrics for survival. Since the COX approach aims at investigating the predictive value of empirical factors only, we also calculated from the observed individual TS data the best percentage change from baseline within 12 weeks (PTR_max_), as a surrogate of antitumor response to gemcitabine.

#### Survival analysis

To be able to compare the PAR approach with the more empirical COX approach, only patients included in the TS model-based analysis of the training set and with complete cases for the screened risk factors were retained for the multivariate survival analysis. For clarity, the resulting data set is referred to as the “reduced-training” set, which includes baseline risk factors, the model-derived PTR at week 2–10 and the empirical PTR_max_ metric for all patients.

Since the PAR approach aims at developing a quantitative tool to further guide trial design, a parametric AFT model was used to relate baseline risk factors and model-derived PTR metrics to survival times. The lognormal and Weibull distributions were evaluated for the distribution of survival times *T*. The model can be expressed as follows:

3 $$\begin{aligned} & T \sim {\text{lognormal}}(\mu ,\sigma ) \\ & {\text{or}} \\ & T \sim {\text{Weibull}}(\alpha ,\lambda ) \\ \end{aligned}$$

where $\mu$ ($- \infty$ to $+ \infty$) and $\sigma$ (0 to $+ \infty$) are the mean (location) and scale parameters, respectively, for the lognormal distribution, and $\alpha$ (0 to $+ \infty$) and $\lambda$ (0 to $+ \infty$) are the shape and scale parameters, respectively, for the Weibull distribution. In order to parameterise the models as AFT models, the covariate effects were tested only on $\mu$ for the lognormal model and on $\lambda$ for the Weibull model. For instance, $\lambda$ was defined as follows:

4 $$\lambda = e^{{\theta_{0} + \theta_{x} \cdot x}}$$

where $\theta_{0}$ is the intercept (typical value) of the natural logarithm of $\lambda$ and $\theta_{x}$ the coefficient (effect) of the variable *x*. This parameterisation ensures that the parameter does not take negative values for any patient or group. We chose to implement the model in the software Stan [24] as it provides an easy way to model censored data and offers flexibility on the input data format and model parameterisation. Diffuse prior distributions were assigned to the model parameters (see example of Stan code in “Appendix 2”). Posterior distributions were approximated using the No-U-Turn sampling algorithm. Selection of an appropriate survival distribution was based on approximate leave-one-out cross-validation (LOO) as computed in the R package loo [26]. Once a base model was selected, covariates were included in a forward way based on LOO estimates and the 95 % credible intervals of the regression coefficients. The ability of the proposed model to describe the data in the reduced-training set (calibration ability) was evaluated by visual comparison of the observed and predicted survival curves. One thousand parameter sets were randomly sampled from the posterior distributions. For each parameter set, individual survival curves were simulated on the basis of patient covariates and were then averaged to derive a population survival curve. The median population survival curve and a 95 % credible interval were then calculated based on the 1000 samples. The observed survival curves [median and 95 % confidence interval (CI)] were derived using Kaplan–Meier estimates [27].

A more frequently used approach to model survival data and identify risk factors is to perform a multivariate CPH regression, as was done for the original training set to screen for potential risk factors. Note that to be able to do a head-to-head comparison of the PAR and COX approaches, we repeated the multivariate CPH regression on the reduced-training set to possibly relate baseline risk factors and the empirical PTR_max_ metric to the survival time. In addition, we also built a CPH model based only on the baseline clinical characteristics of the patients, i.e. excluding TS-related variables. Using a penalised regression like the LASSO method, appropriate estimation of parameter uncertainties is not trivial. Therefore, variable reduction was instead done by backward deletion using a significance level of 0.05. The proportional hazards assumption was evaluated for the remaining variables by incorporation of a time interaction and by visual inspection of the scaled Schoenfeld residuals [28]. The discriminative ability of the model was assessed by computing the concordance probability corrected for the bias due to model optimism [29]. Empirical 95 % CIs around the regression coefficient estimates were calculated from 1000 bootstrap resamples. The analysis was carried out using the R package rms [30].

#### Validation

The performance of the PAR and COX approaches in predicting the survival time of new patients was evaluated by applying the models to the validation set and computing the area under a receiver operating characteristic (ROC) curve at different relevant time points. Only patients with complete cases for the proposed risk factors were retained in the validation set. Each ROC curve was derived using patient survival probabilities at a given time *t* as predictor and patient death status at time *t* as binary outcome. Survival probabilities were predicted on the basis of patient covariates in the validation set. For the AFT model, posterior mean parameters were used to simulate survival probabilities. The 95 % CI for the time-dependent area under a ROC curve (AUROC) was computed on the basis of bootstrapping resampling method. In addition, the integrated AUROC values across all time points were calculated as a global predictive accuracy or discrimination measure, using the R package riskset ROC [31]. Finally, since the follow-up period in the validation set was longer than in the training set, we also check the ability of the PAR approach to extrapolate survival data beyond the training follow-up period, by visual comparison of the observed and predicted survival curves.

## Results

### Baseline risk factors

Among the 31 variables screened for the 271 patients of the training set, ECOG score (0 vs. 1 or 2), ALB, ALP (log-transformed), BSA, BILI, CA19-9 (log-transformed), NEUT (log-transformed) and baseline TS (TS_0_) were retained in the sparse model produced by the LASSO penalised CPH regression that is 8 variables in total. While an increase in most variables is associated with worse prognosis, there is an inverse relationship between ALB and BSA and patient survival times. There is evidence in the literature that cancer of the body and tail of the pancreas are associated with poorer survival than head lesions [6]. In addition, it has been shown that diabetes might affect survival of patient with MPC [8]. Although CLOC and diabetes status were not found to be important risk factors when analysing the training set, these variables were further reassessed on the reduced-training set.

### Tumour size time-series model

**Fig. 1:**
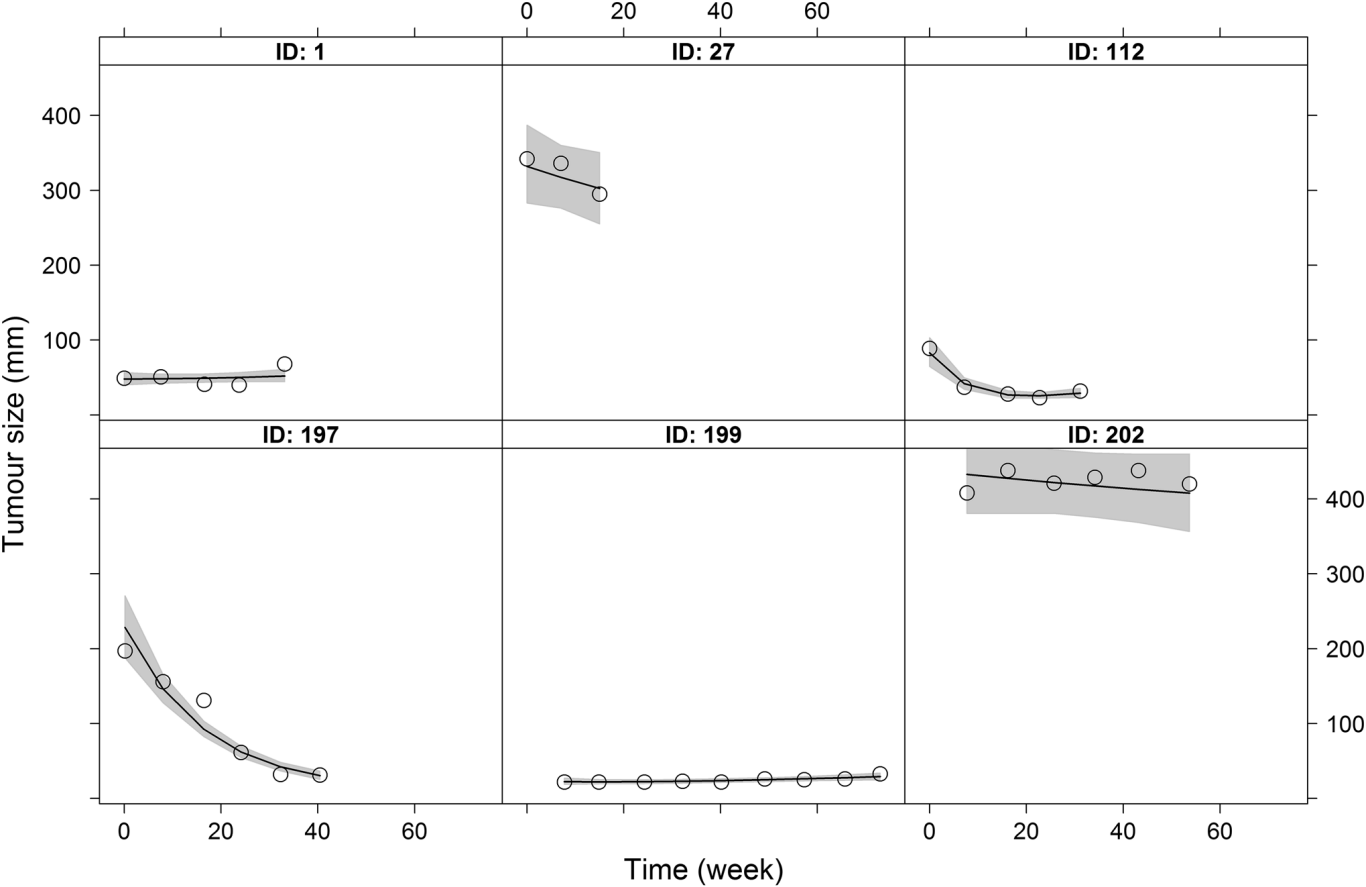
Observed (*black open circles*) and predicted (*solid black lines*) tumour size time course for six representative patients of the training set. The *grey areas* represent 95 % credible intervals for the individual predictions

The hierarchical nonlinear model provided a good description of the median trend and the variability in the TS time-series of both the training and validation sets (Figure S1 in the Online Resource). Of note, the TS model was fitted to the validation data which were used to validate only the survival models. The predicted median trends in the TS time-series suggest that under gemcitabine treatment, tumours (target lesions) typically undergo slight shrinkage followed by smooth regrowth. Although this trend varies across the 152 patients of the training set and across the 385 patients of the validation set (e.g. TS only increasing for some patients), the model was flexible enough to reasonably capture individual profiles (Fig. 1). The posterior distributions of the model parameters are summarised in Table 1. Convergence was considered achieved for all marginal parameter distributions based on the potential scale reduction statistics (all <1.1). The uncertainty in all parameter values was reasonably small as indicated by the standard deviations.

**Table 1:** Posterior quantities for the parameters of the tumour size time-series models for the training and validation sets

Parameter	Training		Validation	
	Mean	SD	Mean	SD
*θ*_BASE_	4.58	0.0414	4.52	0.0367
*θ*_SR_	−6.78	0.166	−6.92	0.0702
*θ*_PR_	−3.17	0.301	−5.79	0.643
*ω*_BASE_	0.648	0.0307	0.682	0.0267
*ω*_SR_	1.32	0.135	1.27	0.0976
*ω*_PR_	0.835	0.226	2.45	0.368
*Σ*	0.150	0.0065	0.235	0.0071

*SD* standard deviation

*Symbols* *θ* log-transformed population means, *ω* interpatient standard deviations, *σ* residual standard deviation

Posterior means and SDs calculated on 2000 samples obtained by pooling 4 chains of 500 samples after burn-in for each

### Survival analysis

Among the 152 patients for whom PTR at week 2–10 could be interpolated and PTR_max_ could be calculated, 120 patients had complete cases for ECOG score, ALB, ALP, BSA, BILI, CA19-9, CLOC, diabetes status, NEUT and TS_0_ (observed) and were therefore retained in the reduced-training set. This data set was used to build a multivariate survival model by application of either the PAR or the COX approach. The distributions of the variables in the reduced-training set are presented in the first column of Table 2. Fifty-eight patients (48 %) had died by the end of the study, with a median follow-up time of 19 weeks for the 62 patients still alive. The median survival time estimate for the studied group is 33 weeks (95 % CI from 29 to 42 weeks).

**Table 2:** Baseline characteristics and tumour size reduction metrics for patients by the reduced-training set (complete cases from the original training set) and the validation set

Variable	Reduced-training set	Validation set
	(120 patients, 58 deaths)	(235 patients, 204 deaths)
Categorical	Category (%)	Category (%)
CLOC	Entire pancreas or head (68) versus body or tail (32)	NE
DIAB	Yes (32) versus no (68)	NE
ECOG	0 (37) versus 1 or 2 (63)	NE

Continuous	Median (range)	Median (range)
ALB (G/l)	41 (26–62)	43 (28–53)
ALP (IU/l)	161 (5.04–1686)	NE
BSA (m^2^)	1.78 (1.31–2.29)	1.84 (1.34–2.49)
BILI (µmol/l)	10.3 (1.71–34.4)	NE
CA19-9 (IU/ml)	1426 (0.6–294,800)	NE
NEUT (G/l)	5.75 (1.55–34.7)	5.40 (1.50–17.3)
TS_0_ (mm)	107 (19–378)	95 (10–358)
PTR (%)^a^		
wk2	1.00 (−5.20 to 25.8)	1.45 (−17.7 to 51.32)
wk4	1.97 (−10.4 to 44.3)	2.87 (−35.4 to 75.7)
wk6	2.89 (−15.7 to 57.4)	4.25 (−53.1 to 87.1)
wk8	3.78 (−21.0 to 66.7)	5.61 (−70.9 to 92.2)
wk10	4.66 (−26.3 to 73.1)	6.94 (−88.7 to 94.4)
PTR_max_ (%)	9.59 (−71.4 to 78.9)	7.14 (−582 to 100)

*NE* not extracted, *CLOC* pancreatic cancer location, *DIAB* diabetes status, *ECOG* Eastern Cooperative Oncology Group status score, *ALB* albumin, *ALP* alkaline phosphatase, *BSA* body surface area, *BILI* bilirubin, *CA19*-*9* carbohydrate antigen 19-9, *NEUT* neutrophil count, *TS*_*0*_ baseline tumour size, *PTR*_*wkx*_ percentage tumour size change from baseline at week *x*, *PTR*_*max*_ best percentage tumour size change from baseline within 12 weeks

^a^Model-derived

#### The PAR approach

The AFT models using a lognormal distribution (AFT_LN_) or a Weibull distribution (AFT_WB_) for the distribution of survival times provided similar performance in describing the reduced-training set as indicated by the LOO estimates (−265.9 and −265.8 for the AFT_LN_ and AFT_WB_ models, respectively) and their standard errors (21.52 and 21.23 for the AFT_LN_ and AFT_WB_ models, respectively). A visual evaluation of the descriptive performance of the models is depicted in Figure S2 in the Online Resource. Hence, both models were used for the multivariate analysis and were further validated. Using the AFT_LN_, TS_0_ (centred at the median), ALB (centred at the median) and PTR at week 2 (PTR_wk2_) were found to be the best predictor of patient time to death. When analysing the survival data with the AFT_WB_ model, only TS_0_ and BSA (centred at the median) were identified as predictors. It should be noted that the predictive values of PTR at week 2, 4, 6, 8 and 10 were the same when using the AFT_LN_ model. Nevertheless, we chose to carry on the analysis with the earliest TS reduction metric, i.e. PTR at week 2. In the AFT_WB_ model, the coefficients of all PTR variables have credible intervals that include zero. An example of Stan code for the AFT_WB_ model is presented in “Appendix 2”. Potential scale reduction statistics (all ˂1.1) indicated that all parameter distributions seemed to have converged to approximate equilibrium. The posterior marginal distributions are summarised in Table 3.

**Table 3:** Posterior quantities for the parameters of the lognormal and Weibull accelerated failure time models for the survival data of the reduced-training set

Parameter	Mean	95 % credible interval
Lognormal		
log(*µ*)		
*θ*_0_	1.25	(1.21; 1.29)
*θ*_TS0_	−0.001	(−0.0015; −0.0005)
*θ*_PTRwk2_	1.07	(0.189; 2.01)
*θ*_ALB_	0.0079	(0.0006; 0.0151)
*σ*	0.544	(0.453; 0.661)
Weibull		
*α*	2.33	(1.92; 2.75)
log(*λ*)		
*θ*_0_	3.80	(3.67; 3.93)
*θ*_TS0_	−0.0037	(−0.0052; −0.0022)
*θ*_BSA_	0.762	(0.103; 1.44)

Lognormal distribution: *µ* is the mean and *σ* the scale parameter

Weibull distribution: *α* is the shape and *λ* the scale parameter

Intercepts *θ*_0_ and coefficients *θ*_*x*_ are for the log-transformed parameters that include covariates (see “Material and methods” and example of Stan code in “Appendix 2”)

Posterior means and percentiles were calculated on 2000 samples obtained by pooling 4 chains of 500 samples after burn-in for each

#### The COX approach

After backward deletion of the 11 variables (including the TS_0_ and PTR_max_ variables) evaluated in the multivariate CPH model, only TS_0_ (centred at the median), BSA (centred at the median) and PTR_max_ were found to be significant risk factors (*P* values <0.0001, of 0. 0087 and of 0.0008, respectively). However, the bootstrap 95 % CI for the BSA coefficient included zero (−3.97 to 0.485). Hence, only TS_0_ (in mm) and PTR_max_ were retained in the CPH model (referred to as the “COX_1_” model) with coefficient estimates of 0.0064 (95 % CI from 0.0032 to 0.0134) and −1.29 (95 % CI from −4.40 to −0.184), respectively. Interaction of these two variables with time did not significantly improve the model (all *P* values >0.2) which suggests that the assumption of proportional hazards is reasonable (see also Figure S3 in the Online Resource). When excluding the TS-related variables TS_0_ and PTR_max_ from the multivariate regression, only NEUT (log-transformed) was identified as significant survival predictor (*P* = 0.0006) with coefficient estimate of 0.759 (95 % CI from 0.336 to 1.72). The interaction with time also did not significantly improve the model (*P* > 0.1). This model is referred to as the “COX_2_” model in the rest of the report. The bias-corrected concordance probability was estimated to be 0.71 and 0.66 for the COX_1_ and COX_2_ models, respectively, which suggests that the COX_1_ model distinguishes high-risk patients from low-risk patients better than the COX_2_ model.

### Predictive performance

Only patients with complete cases for ALB, BSA, NEUT, PTR_wk2_, PTR_max_ and TS_0_ were retained in the validation set (235 patients, 204 deaths) to evaluate the performance of the PAR and the COX approaches in predicting MPC survival data. The distribution of these variables is summarised in the second column of Table 2. The median follow-up time for patients still alive was 57 weeks, and the median survival time estimate for the group is 35 weeks (95 % CI from 30 to 40 weeks).

**Fig. 2:**
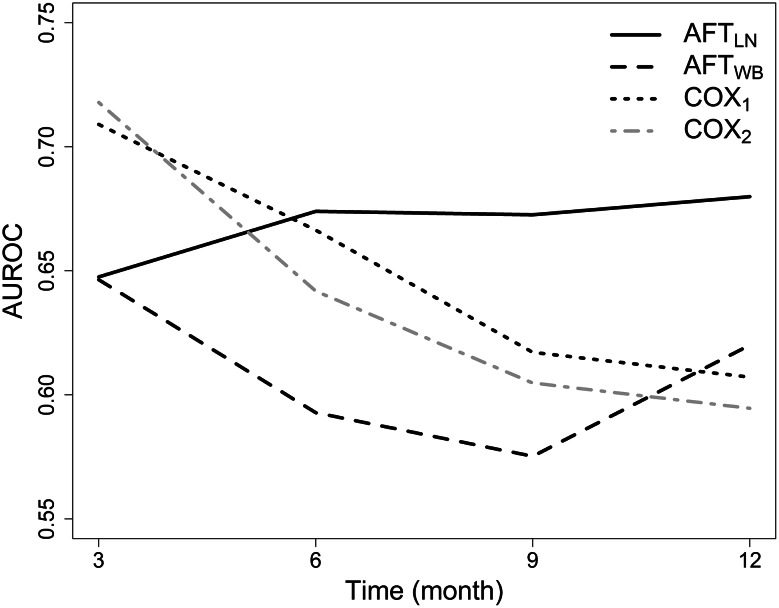
Time-dependent area under the ROC curve for the two parametric accelerated failure time models evaluated by the PAR approach and for the two Cox proportional hazards models evaluated by the COX approach

**Fig. 3:**
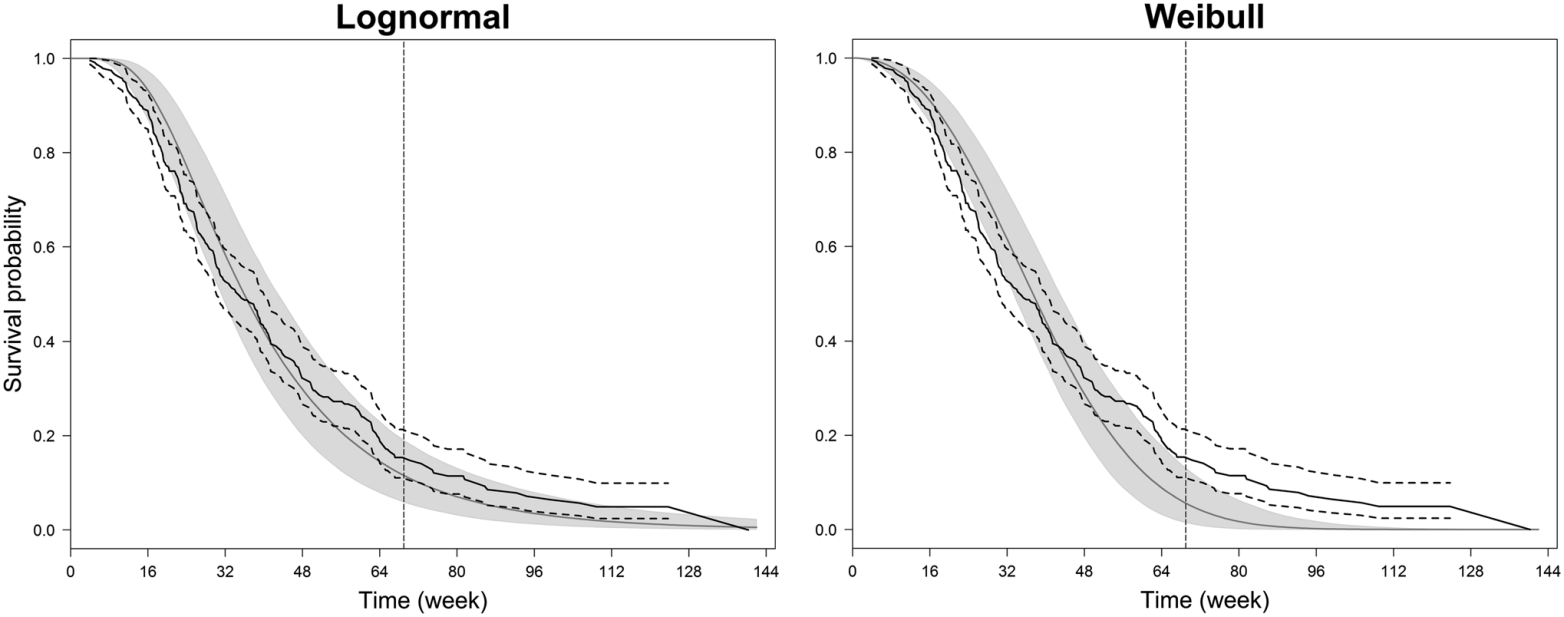
A visual evaluation of the ability of the lognormal and Weibull accelerated failure time models to predict the survival data in the validation set. The observed median survival curves (*solid black lines*) are plotted along with their 95 % confidence intervals (*dashed black lines*) as well as with the simulated median survival curves (*solid grey lines*) and their 95 % credible intervals (*grey areas*). The *vertical dashed lines* represent the last follow-up time in the reduced-training set, i.e. 69 weeks

The predictive performance of the two approaches was compared by calculating the AUROC at 3, 6, 9 and 12 months. This time points were chosen because the median survival times reported in recent randomised trials [19, 20] fall in this range. The time dependency of AUROC for the AFT_LN_, AFT_WB_, COX_1_ and COX_2_ models is depicted in Fig. 2. The AFT_LN_ model typically performs better than the other models. Also, the performance of the COX models seems to decrease with time. However, for clarity, the AUROC time curves are plotted without their confidence bands which all overlap at any time. The integrated AUROC values across all time points were 0.68 (95 % CI 0.60–0.75), 0.60 (95 % CI 0.51–0.68), 0.65 (95 % CI 0.57–0.73) and 0.64 (95 % CI 0.55–0.72) for the AFT_LN_, AFT_WB_, COX_1_ and COX_2_ models, respectively. The 95 % CIs of the integrated AUROC values overlap, which suggests that all four models perform globally, similarly on the validation set. The extrapolation ability of the AFT_LN_ and AFT_WB_ models is illustrated in Fig. 3 and seems to be higher for the AFT_LN_.

## Discussion

Prognostic models for survival of patients with MPC are essential to identify stratification variables and control for known important variability in the data when conducting large, prospective, Phase III randomised controlled trials. It is also essential to individualise care and treat patients more effectively. In this study, two different survival modelling approaches have been evaluated retrospectively using the control arm data of two independent Phase III studies of patients under gemcitabine treatment. The first approach, referred to as the PAR approach, aims at incorporating model-predicted TS reduction metrics into a parametric survival model that can be used for clinical trial simulations. This approach utilises time-series of imaging data to interpolate early change in TS for all patients. The second approach, referred to as the COX approach, simply aims at identifying empirical prognostic factors with the commonly used multivariate CPH regression model. Our results suggest that the two approaches perform similarly in predicting survival probability of new MPC patients, as indicated by the 95 % CIs of the integrated AUROC values.

Regardless of the modelling approach applied, the baseline variable TS_0_ appears to be a significant prognostic factor for patients with MPC, although the effect is weak overall: in the COX_1_ model for instance, the hazard ratio (HR) for 1-cm increase in TS_0_ is 1.07 (95 % CI 1.03–1.14); in the AFT_LN_ model, the ratio of median survival time (AF) for 1-cm increase in TS_0_ is 0.990 (95 % credible interval 0.985–0.995). The other risk factors identified were different depending on which approach was applied. With the PAR approach, the metric PTR at week 2 was identified as strong survival predictors in the AFT_LN_ model (AF ratio of 2.92 for 1 unit increase, with 95 % credible interval from 1.21 to 7.46) although it was not in the AFT_WB_ model. The fact that the predictive value of the PTR metric was similar at each time point assessed (week 2–10) is consistent with the predicted median TS-dynamic trend in the studied patients treated with gemcitabine (Figure S1 in the Online Resource). Also, patients with higher albumin levels were found to survive slightly longer (AF ratio of 1.08 with 95 % credible interval from 1.01 to 1.16), which is consistent with biology (inflammation marker) and with a previously reported MPC prognostic model [7]. Finally, increase in BSA was related to increase in median survival time (AF ratio of 2.14 with 95 % credible interval from 1.11 to 4.22) in the AFT_WB_ model. Since patients undergoing gemcitabine chemotherapy are dosed by BSA, this means that patients with higher doses might survive longer, which suggests that the standard dose for gemcitabine (1000 mg/m^2^) might not be optimal for all studied MPC patients. Using the COX approach, PTR_max_ was identified as significant risk factor with HR estimate of 0.275 (95 % CI 0.0123–0.832). However, the CPH model that includes only NEUT as covariate (COX_2_ model) performed as well as the model with both TS_0_ and PTR_max_ (COX_1_ model) in predicting patient survival, thereby suggesting that variables derived from TS imaging data might not be better prognostic factors for patients with MPC than some of the toxicology markers like baseline neutrophils. Patients with high pre-treatment neutrophil counts are probably more at risk than patients with lower counts (HR ratio of 2.14 with 95 % CI from 1.40 to 5.58), as it was suggested in a previous study [10]. It should be noted that only NEUT was retained in the CPH model when analysing the reduced-training set (excluding TS-related variables) compared to 8 variables when analysing the original training set using the LASSO method. This can be explained by the decreased power of the analysis when reducing the training set and probably also by the difference of variable selection method.

The choice of the modelling approach clearly depends on the purpose of the survival analysis. The PAR approach has been recommended to aid oncology drug development decisions such as compound screening, dose selection and trial design [15–18]. In the present example for MPC, this approach could reasonably predict the mortality risk of patients from an independent study, with similar performance compared to the more conventional COX approach. However, our work suggests caveats against the PAR approach. Firstly, a unique AFT model could not be selected on the basis of the training set (see Figure S2 in the Online Resource), possibly due to the small study sample size (120 patients, 58 deaths). This emphasises the difficulty of defining the distribution of survival times when using parametric models with limited survival data. Although the AFT_LN_ and AFT_WB_ models could predict the validation data with similar global predictive accuracy, the distributional assumption on time to death seems to affect the identification of prognostic factors (Table 3) as well as the extrapolation ability of the model (Fig. 3). It should be stressed that using the Weibull distribution, longitudinal TS metrics were not identified as better survival predictors than other baseline characteristics. Nevertheless, we acknowledge that other commonly used AFT models, such as the log-logistic and generalised gamma distributions, were not assessed in this analysis and might provide a better description of the training data than the lognormal and Weibull distributions. Secondly, the PAR approach is more time-consuming than the COX approach as it involves modelling the TS dynamics rather than simply evaluating empirical metrics. Also, the individual model parameters, used to interpolate early change in TS for each patient, always carry uncertainty. For simplicity, we ignored parameter uncertainty in the present analysis by using the posterior means of the individual parameters. However, this can introduce bias in the subsequent evaluation of these variables as survival predictors. Ideally, several sets of TS reduction metrics should be produced by sampling from the posterior distribution of the individual parameters, if a Bayesian approach is applied. This issue has also been addressed in a frequentist modelling approach where shrinkage of individual parameter estimates can affect the type I error of falsely detecting or failing to detect TS metrics as predictors of survival [32].

We developed the parametric AFT models using a Bayesian approach mainly because we found a probabilistic programming language convenient for the analysis of censored data and because credible intervals for the covariate effects are obtained. The identification of prognostic factors was simply done by forward selection based on LOO estimates and the 95 % credible intervals of the coefficients. Alternatively, methods that use shrinkage priors could be employed for variable selection [33, 34]. These methods are similar to frequentist penalised regressions, in the sense that the coefficients of (apparently) irrelevant covariates would have credible intervals that include zero, although it offers the advantage of readily producing the uncertainty distribution of the parameters.

In conclusion, the PAR modelling approach that utilises model-derived TS metrics in addition to baseline patient characteristics could predict reasonably well survival of patients with MPC undergoing gemcitabine chemotherapy. However, determining the distribution of survival times appeared challenging with data from only one small study and seems to affect the identification of risk factors. Moreover, the predictive performance was not significantly better than a simple CPH model that incorporates only baseline neutrophil count as covariate. Nevertheless, our findings should be confirmed by analysing data sets that have higher power for multivariable survival regression. In particular, the predictive value of the new potential prognostic factors BSA (gemcitabine dose) and TS-related metrics should be reassessed together with established risk factors on a larger MPC study.

### Electronic supplementary material

Below is the link to the electronic supplementary material.

Supplementary material 1 (DOCX 379 kb)
