# Supplementary material for: Predicting survival of pancreatic cancer patients treated with gemcitabine using longitudinal tumour size data
Source: Cancer Chemother Pharmacol. 2016 Mar 3;77:927–38. doi: 10.1007/s00280-016-2994-x (PMC4844653; doi:10.1007/s00280-016-2994-x)
Supplement: Supplementary file 1 — Supplementary material 1 (DOCX 379 kb) [file 280_2016_2994_MOESM1_ESM.docx]

Supplementary material for the manuscript “Predicting survival of pancreatic cancer patients treated with gemcitabine using longitudinal tumour size data” submitted to the journal

Cancer Chemotherapy and Pharmacology

**T Wendling^1, 2^, H Mistry^1^, K Ogungbenro^1^ and L Aarons^1^**

^1^ Manchester Pharmacy School, The University of Manchester, M13 9PT, Manchester, United-Kingdom.

^2^ Drug Metabolism and Pharmacokinetics, Novartis Institutes for Biomedical Research, 4056, Basel, Switzerland.

**Corresponding author:**

Thierry Wendling

Address: Stopford Building Room 3.32, Oxford Road, Manchester, M13 9PT

E-mail: [thierry.wendling@manchester.ac.uk](mailto:thierry.wendling@manchester.ac.uk)

Phone: +44 161 275 7105

Fax: +44 161 275 8349


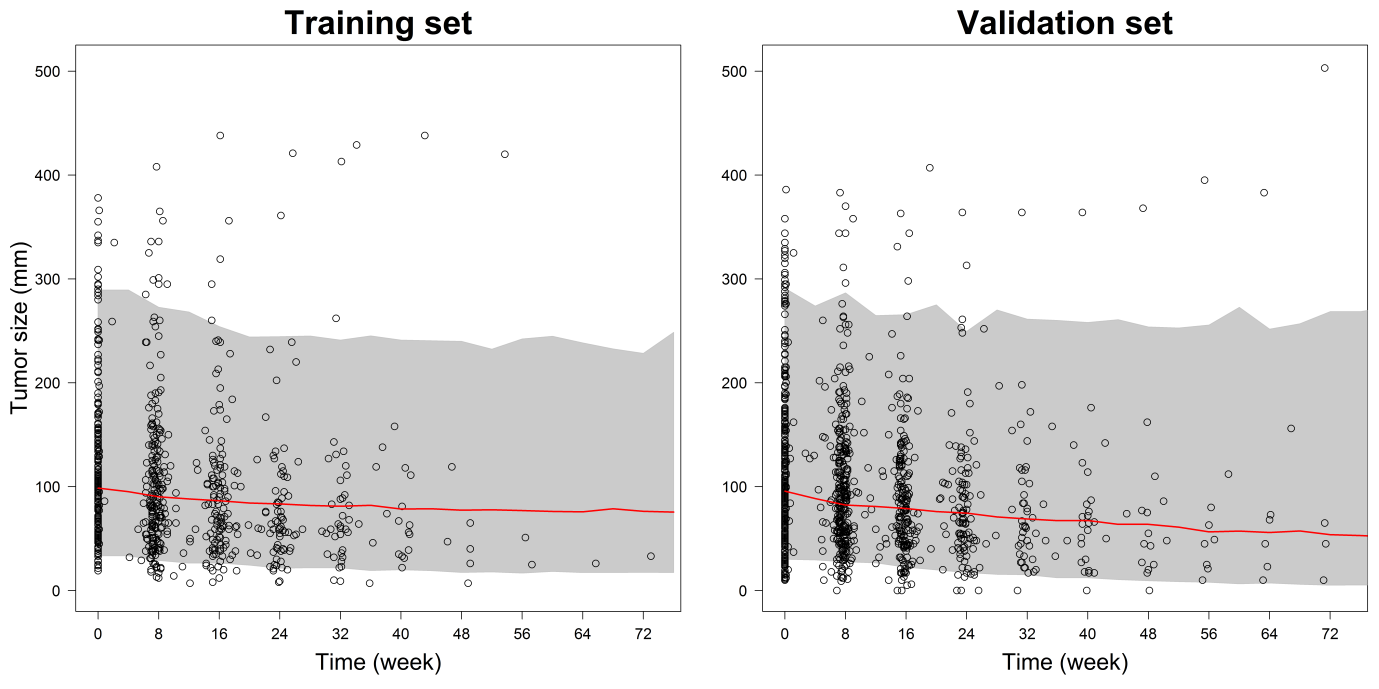


**Figure S1** A visual evaluation of the ability of the tumour size time-series model to describe the training and validation data. The *black open circles* are the observations, the *red lines* are the medians of the simulations and the *grey areas* are 90% prediction intervals


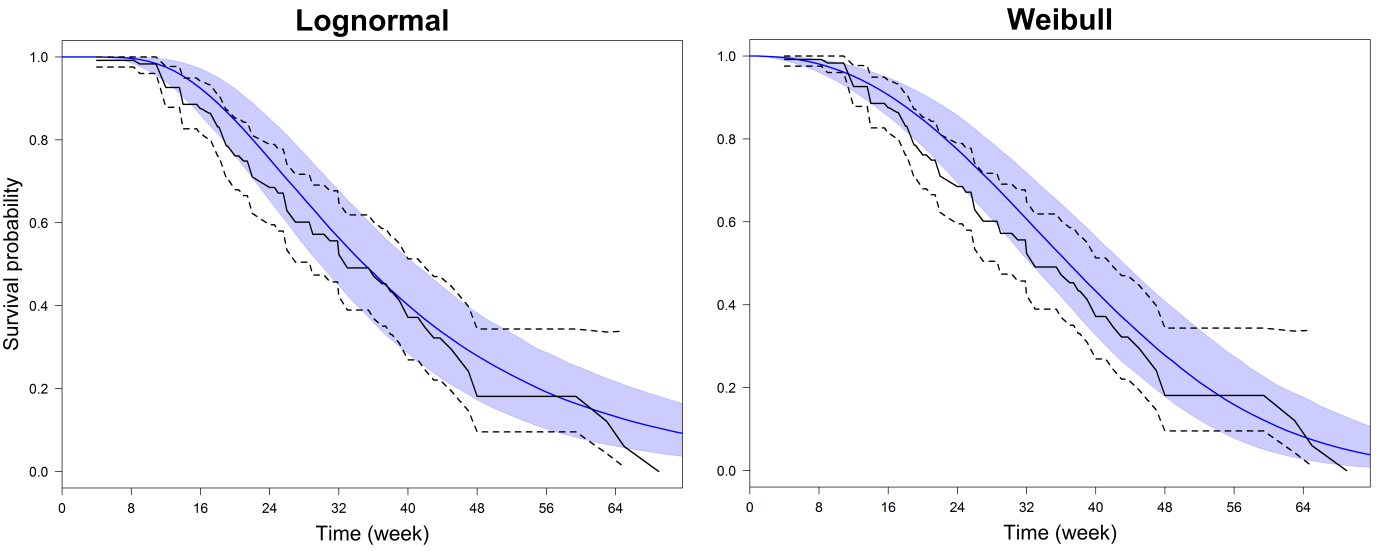


**Figure S2** A visual evaluation of the ability of the lognormal and Weibull accelerated failure time models to describe the survival data in the reduced-training set. The observed median survival curves (*solid black lines*) are plotted along with their 95% confidence intervals (*dashed black lines*) as well as with the simulated median survival curves (*solid blue lines*) and their 95% credible intervals (*blue areas*)


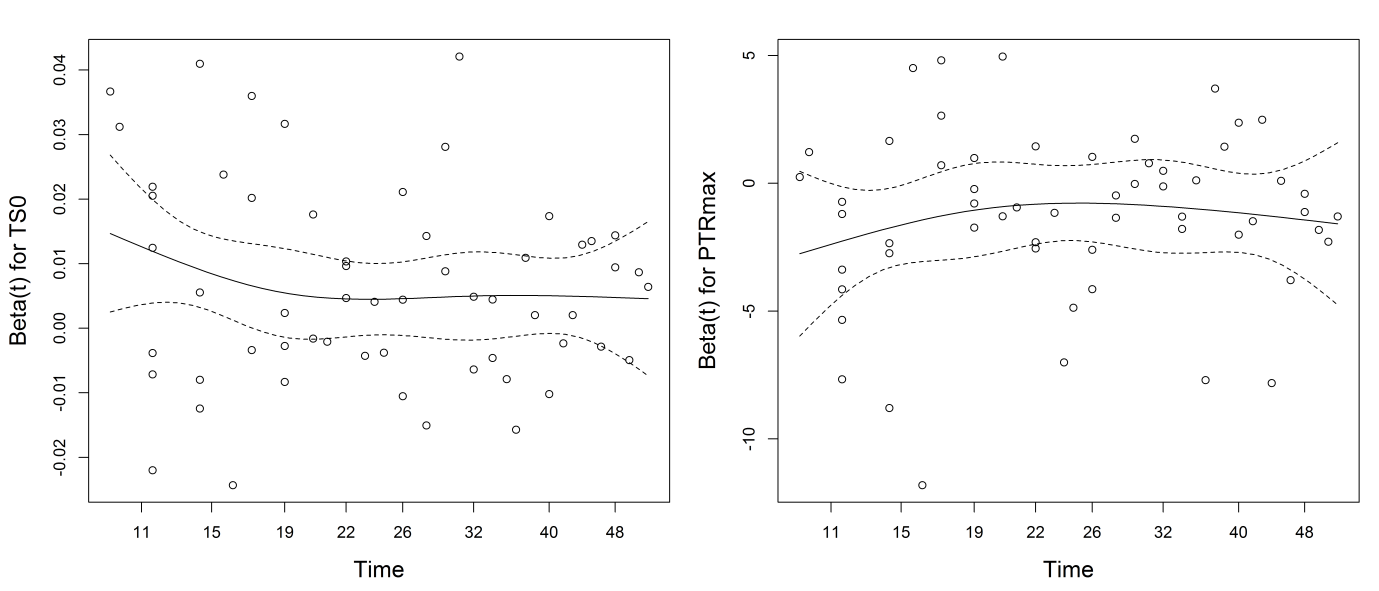


**Figure S3** Scaled Schoenfeld residuals (*open circles*) plotted against time for the two variables retained in the COX_1_ model, namely TS_0_ and PTR_max_. The *solid lines* represent fitted natural splines and the *dashed lines* their 95% confidence intervals
